# Supplementary material for: ArsenicNet: An efficient way of arsenic skin disease detection using enriched fusion Xception model
Source: PLoS One. 2025 May 30;20(5):e0322405. doi: 10.1371/journal.pone.0322405 (PMC12124517; doi:10.1371/journal.pone.0322405)
Supplement: Attachment — Submitted filename: Arsenic_PLOS ONE-Response-to-Reviewers 2.pdf [file pone.0322405.s002.pdf]

Original Manuscript ID: PONE-D-24-44589R1

Original Article Title: **“**ArsenicNet: An Efficient Way of Arsenic Skin Disease Detection Using Enriched Fusion Xception Model”

To: PLOS ONE Editor

Re: Response to reviewers

Dear Editor,

Thank you for allowing a resubmission of our manuscript, with an opportunity to address the reviewers’ comments.

We are uploading (a) our point-by-point response to the comments (below) (response to reviewers, under “Author’s Response Files*”*), (b) an updated manuscript with yellow highlighting indicating changes (as “Highlighted PDF*”*), and (c) a clean updated manuscript without highlights (“Main Manuscript”*).*

Best regards,

M.H.K. Mehedi et al.

Reviewer#2: I commend the authors for their effort in improving the manuscript based on the previous comments, but I still believe there are still a few things to address.

Reviewer#2, Concern # 1 (please list here): Language and Structure: Although the authors indicated that the manuscript has been proofread, some sections still require general language and structural editing for improved readability. For example, in the abstract, a statement like "The proposed model achieved 97.69% accuracy, along with 97.63% F1 score and reached the top"; in section 4.3, a statement like "More over, if we notice closely, then we can see that we have got the lowest test accuracy,..."; etc.

Recommendation: Professional language editing will perhaps be much better.

Author response: Thank you for the comment.

Author action: We have revised the manuscript and proofread it by a native speaker.

Reviewer#2, Concern #2(please list here): The figure illustrating the model architecture needs clarity. Adequate annotations explaining each component and its role in the architecture are important.

Author response: Thank you for the suggestion.

Author action: We have clearly drawn and annotated the model architecture figure. We have also provided a detailed description of each component's role.

Reviewer#2, Concern #3(please list here): Dataset size: The author acknowledged that the dataset is small. This is a big concern as to the generalisability of the model findings.

Recommendation: The author should include a comprehensive discussion (also in the abstract) on the limitations of the research (particularly the chances of model overfitting).

Author response: Thank you for the feedback.

Author action: As we already said, the dataset is small in size and there are no alternative datasets available. So, there might be a chance of model overfitting. To prevent this issue, we applied different data augmentation techniques, such as horizontal flip, width, and height shift. In addition, we used early stopping function to prevent overfitting issues by monitoring val_loss.
